# Accessory gallbladder lithiasis presenting as acute cholangitis

**DOI:** 10.1016/j.radcr.2026.07.110

**Published:** 2026-08-27

**Authors:** Houssem Hamrouni, Hatem Hamrouni, Faten El Hedhili, Mohamed Mahmoud Vall, Aymen Merdassi, Ivelina Choneva

**Affiliations:** Radiology Department, Sud Ile de France Hospital, Melun, France

**Keywords:** Accessory gallbladder, Acute cholangitis, Common bile duct stone, MRCP

## Abstract

Accessory gallbladder is an uncommon congenital anomaly that may present significant diagnostic and surgical challenges, particularly when associated with biliary complications. We report a case presenting features of common bile duct obstruction and cholangitis. Multimodality imaging, including computed tomography and magnetic resonance cholangiopancreatography, enabled preoperative identification of an accessory gallbladder. Magnetic resonance cholangiopancreatography was especially useful in differentiating this anomaly from other cystic biliary lesions and in accurately delineating the biliary anatomy. These findings guided management, allowing detection of common bile duct stone migration and appropriate treatment. This case highlights the key role of imaging in the accurate diagnosis of rare biliary anomalies and in reducing the risk of surgical complications*.*

## Introduction

Accessory gallbladder is a rare biliary tract anomaly related to abnormal organogenesis, occurring with an estimated incidence of 1 in 4000 to 5000 cases [1]. A higher prevalence has been observed in females compared to males.

While accessory gallbladder is typically asymptomatic, we herein report a case discovered incidentally during the management of acute cholangitis. In the vast majority of cases the diagnosis of accessory gall bladder is made during surgery due to the lack of sensitivity of ultrasound.

## Case presentation

A 36-year-old male was admitted to the emergency department presenting with abdominal pain, fever, and jaundice. He had no significant past medical history. Laboratory investigations revealed a cholestatic pattern with associated cytolysis, including elevated total bilirubin (145 µmol/L; institutional reference range: 3-21 µmol/L), direct bilirubin (115 µmol/L; institutional reference range: <5 µmol/L), alkaline phosphatase (612 U/L; institutional reference range: 40-130 U/L), gamma-glutamyl transferase (785 U/L; institutional reference range: 10-60 U/L), alanine aminotransferase (ALT, 324 U/L; institutional reference range: 7-55 U/L), and aspartate aminotransferase (AST, 281 U/L; institutional reference range: 8-48 U/L), consistent with biliary obstruction due to acute calculous cholangitis.

**Fig. 1:**
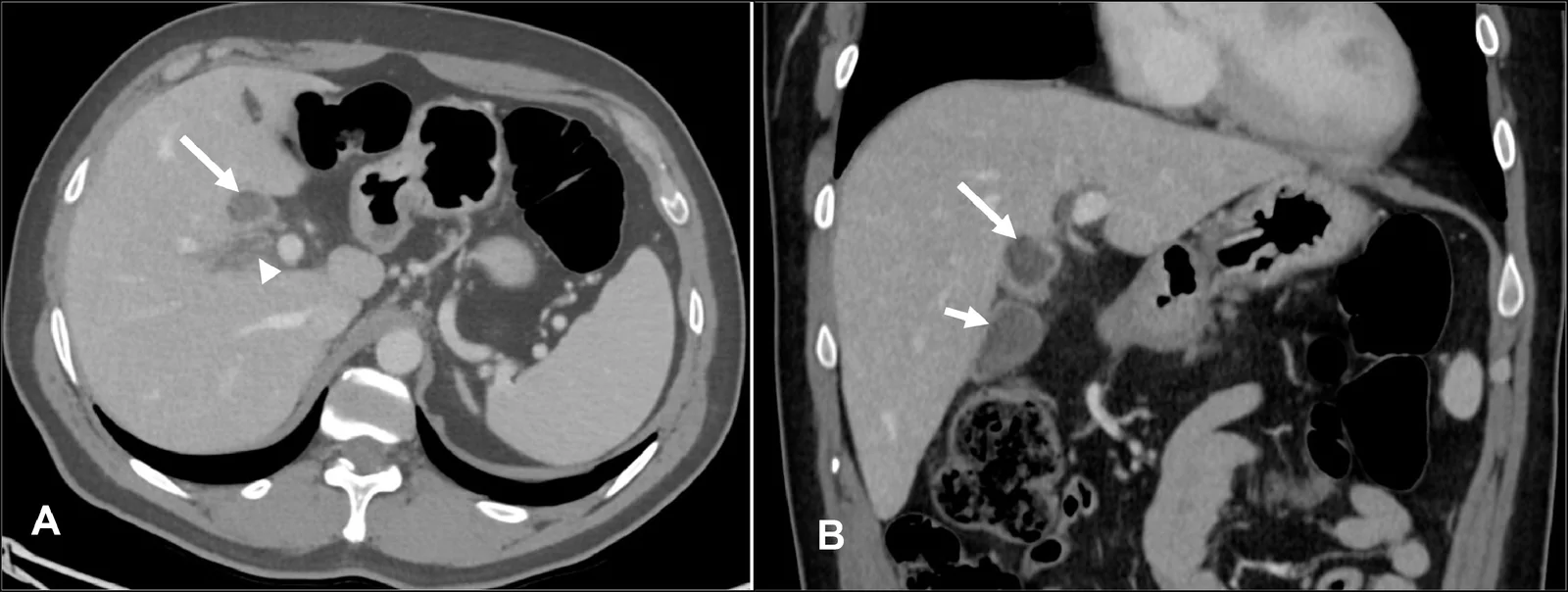
(A and B) Axial and coronal contrast-enhanced CT images demonstrate an ovoid structure, the accessory gallbladder (long white arrow) located superior to the orthotopic gallbladder (short arrow). The common bile duct is dilated, with wall thickening and enhancement, consistent with acute cholangitis (arrowhead).

Abdominal CT (Fig. 1) demonstrated an ovoid structure superior to the gallbladder fossa and biliary duct dilatation with associated mild wall enhancement.

**Fig. 2:**
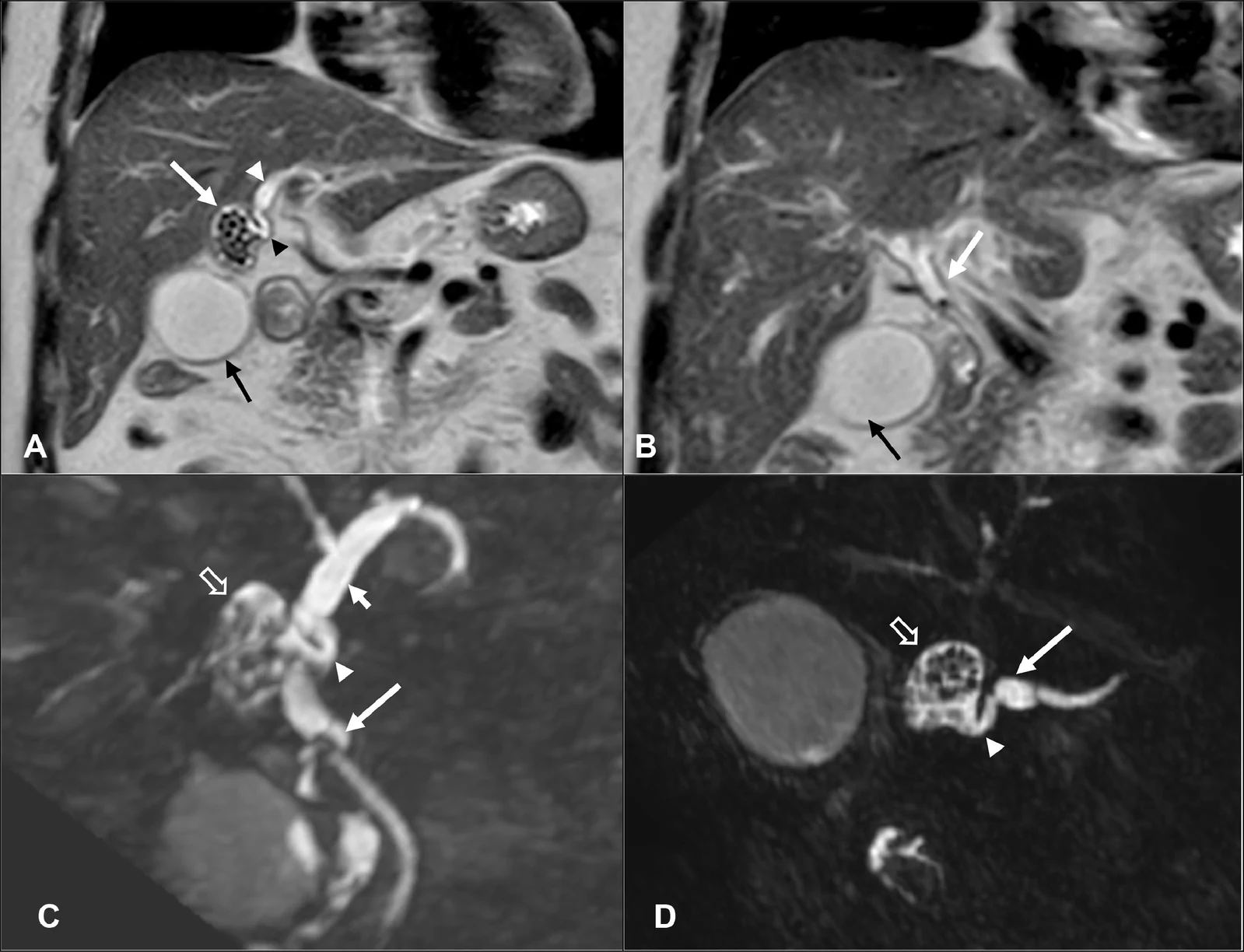
(A) Axial T2 TSE weighted image demonstrates an accessory gallbladder containing multiple microlithiasis (white arrow), with communication to the left hepatic duct (white arrowhead) via an independent cystic duct (black arrowhead), orthodromic gallbladder (black arrow). (B) Coronal T2 TSE weighted image demonstrates biliary dilation due to stone migration (white arrow), orthodromic gallbladder (black arrow). (C) Coronal breath-hold 3D T2 TSE MRCP shows the accessory gallbladder (open arrow), accessory bile duct (arrowhead) connected to the left hepatic duct (short arrow) and biliary dilatation secondary to stone migration into the common bile duct (white arrow). (D) Axial breath-hold 3D T2 TSE MRCP showing accessory gallbladder (open arrow), accessory cystic duct (arrowhead), left hepatic duct (white arrow).

Magnetic resonance cholangiopancreatography (Fig. 2) demonstrated that this structure represented a cystic structure containing microlithiasis, without wall thickening, abnormal enhancement, or surrounding inflammatory changes. It was connected to the left hepatic duct immediately proximal to the biliary confluence by an independent cystic duct, consistent with a type II accessory gallbladder according to Harlaftis classification and an H-type duplication according to Boyden’s classification. It also demonstrated intrahepatic and common bile duct dilatation, with biliary wall contrast enhancement consistent with cholangitis, secondary to an obstructing migrated microlithiasis within the mid portion of the common bile duct.

The patient was subsequently transferred to the gastroenterology department for further evaluation, where an endoscopic retrograde cholangiopancreatography (ERCP) was performed for stone extraction.

The patient then underwent laparoscopic surgery, during which the diagnosis of accessory gallbladder with an independent cystic duct connected to the left hepatic duct was confirmed. Both the accessory and orthodromic gallbladder were resected. The postoperative course was uneventful, and the patient was discharged a few days later. Histopathological examination confirmed the diagnosis.

## Discussion

Between the fourth and fifth weeks of embryonic development, an endodermal diverticulum arising from the anterior primitive foregut gives rise to the gallbladder and cystic duct. An abnormal outgrowth of a secondary diverticulum, referred to as the rudimentary bile bud, may result in the formation of accessory structures along the extrahepatic biliary tree. Depending on the site of its development, this anomalous budding can give rise to an accessory gallbladder [2,3]. Harlaftis classification is the most commonly used, it includes 3 types, Type I: split primordial gallbladder, Type II: 2 separate gallbladders with their own cystic ducts and Type III: triple gallbladders drained by 1-3 separate cystic ducts [4]. Alternatively, Boyden’s classification describes 3 subtypes. A bilobed gallbladder is characterized by a single organ divided into 2 lobes within the same fossa. H-type, the most common variant, consists of 2 separate gallbladders with cystic ducts draining independently into the biliary tree. Y-type, both gallbladders share a common cystic duct that drains into the common bile duct [5].

Patients with accessory gallbladder are typically asymptomatic. However, when complications occur, they present with right upper quadrant pain similar to that observed in common biliary conditions such as cholelithiasis and cholecystitis. Cholangitis and pancreatitis may also develop in cases of stone migration and manifest with clinical features comparable to those seen in individuals with a single gallbladder.

Ultrasound has particularly low sensitivity for diagnosing double gallbladders. CT scan and MRCP are more accurate for characterizing the anatomy and identifying both gallbladders and their cystic duct anatomy especially 3D cholangiopancreatography sequence [6]. In a significant proportion of cases, this abnormality is discovered incidentally during surgery. Failure to recognize it may increase the risk of bile duct injury, as well as postoperative recurrence of gallstones and cholecystitis.

Cystic lesions adjacent to the primary gallbladder may mimic accessory gallbladder, particularly Todani II choledochal cyst [7,8]. Differentiation between these entities can be challenging on preoperative imaging, and definitive diagnosis often relies on intraoperative findings and histopathological examination. Other conditions should also be considered in the differential diagnosis, including perivesicular fluid collections and folded gallbladder. MRCP is usually very useful in differentiating between these diagnoses.

In our instance, the laboratory results and clinical presentation pointed to a common bile duct obstruction complicated by cholangitis, an uncommon situation that has only been documented in 9% of cases.

Multimodal imaging, such as CT and MRCP, was successfully used to identify an accessory gallbladder prior to surgery. This is significant because over 50% of cases remain undiagnosed before surgery, and MRCP has a higher sensitivity (97%) than ultrasonography (66%) [7].

Surgical removal of both gallbladders is recommended to minimize the risk of cholelithiasis and recurrent biliary symptoms in the remaining accessory gallbladder. In most cases a laparoscopic surgery is performed. An open approach may be preferred in cases of a high cystic duct insertion to facilitate safe dissection and reduce the risk of biliary injury. Preoperative imaging is essential for accurate delineation of the biliary anatomy, thereby minimizing intraoperative and postoperative complications, including iatrogenic bile duct injury, hepatic vascular injury, and incomplete resection of either the orthotopic or accessory gallbladder [[9], [10], [11]].

## Conclusion

Accessory gallbladder is a rare entity that requires accurate preoperative diagnosis. MRCP plays a key role in differentiating biliary anomalies and guiding appropriate management to prevent surgical complications.

## Declaration of generative AI and AI-assisted technologies in the manuscript preparation process

During the preparation of this work, the author used artificial intelligence (AI) tools, to assist in the drafting and linguistic refinement of this manuscript. These tools were used solely to improve clarity, grammar, and overall readability. All scientific content, interpretations, and conclusions presented in this work are the sole responsibility of the authors.

## Patient consent

Written informed consent was obtained from the patient for publication of this case report and any accompanying images. The patient was informed that personal information would remain confidential and that no identifying details would be published. A copy of the written consent is available for review by the Editor-in-Chief of this journal upon request.

## References

[1] Causey M.W., Miller S., Fernelius C.A., Burgess J.R., Brown T.A., Newton C. (2010). Gallbladder duplication: evaluation, treatment, and classification. J Pediatr Surg.

[2] Ma X.S., Feng M.F., Ke S., Yang L. (2024). Duplicate gallbladders misdiagnosed as residual cholecystitis: a case report and review of the literature. Medicine (Baltimore).

[3] Soret P.A. (2025). POST’U 2025.

[4] Zhou D.K., Huang Y., Kong Y., Ye Z., Ying L.X., Wang W.L. (2020). Complete laparoscopic cholecystectomy for a duplicated gallbladder. Medicine (Baltimore).

[5] Sangwan A., Bladuell G., Kedar R. (2024). Accessory gallbladder with partial encasement by esophageal adenocarcinoma: a rare case report. Radiol Case Rep.

[6] Ye Y.Q., Liang Q., Li E.Z., Gong J.L., Fan J.M., Wang P. (2023). 3D reconstruction of a gallbladder duplication to guide laparoscopic cholecystectomy: a case report and literature review. Medicine (Baltimore).

[7] Darnis B., Mohkam K., Cauchy F., Cazauran J.B., Bancel B., Rode A. (2018). A systematic review of the anatomical findings of multiple gallbladders. HPB (Oxford).

[8] Ouaïssi M., Kianmanesh R., Belghiti J., Ragot E., Mentha G., Adham M. (2015). Todani type II congenital bile duct cyst: European multicenter study of the French Surgical Association and literature review. Ann Surg.

[9] Picchi E., Leomanni P., Dell'Olio V.B., Pucci N., Di Giuliano F., Ferrazzoli V. (2023). Triple gallbladder: radiological review. Clin J Gastroenterol.

[10] Gigot J.F., Van Beers B., Goncette L., Etienne J., Collard A., Jadoul P. (1997). Laparoscopic treatment of gallbladder duplication: a plea for removal of both gallbladders. Surg Endosc.

[11] Ott L., O'Neill J., Cameron D., Callahan M.J., Grover A., Fox V.L. (2022). Triple gallbladder with heterotopic gastric mucosa: a case report. BMC Pediatr..

